# A Clinical Pilgrimage in War Time

**Published:** 1915-10-02

**Authors:** 


### A CLINICAL PILGRIMAGE IN WAR-TIME.

### The Children's Hospitals of Europe.

Dr. Jno. A. Colliver, of Los Angeles, has recently paid a visit to the clinics of the children's hospitals of Europe, and he records his impressions of these in the August number of the *California State Journal of Medicine*. Commencing with London he remarks that here are to be seen children more poorly dressed, poorly nourished, and poverty-stricken than in any other city. The amount of material at the out-patient department of the Great Ormond Street Hospital is so abundant and the assistants so few that examinations are apt to be hurried and superficial. But the opportunities for clinical study are excellent. Especially is this true of rheumatism and of rickets. In reference to the former of these the frequency of subcutaneous nodules impressed Dr. Colliver, for apparently these nodules are rarely seen in America. Other points noted are the removal of the tonsils by enucleation and the value of the clinical demonstrations in the Children's Section of the Royal Society of Medicine. The summing up, however, is rather cruel—" on the whole English physicians are not so accurate in their diagnosis and have a tendency to use more drugs than in other places."

Of Paris the record is confined to a remark on the low morality of the view which is taken of such questions as illegitimacy, prostitution, and so on, and the fact that a girl who has left her situation to give birth to a child may be later on restored to her former position is evidently regarded as a shocking occurrence. This may be, but its record does not give us much information relative to the study of the diseases of children in Paris. Possibly the war has temporarily disorganised this study, and has left the superior morality of the States all the more shining by contrast.

### German and Austrian Clinics.

In the German cities great emphasis is placed on hygiene and prophylaxis. At Pfundler's clinic at Munich doctors from many countries were seen, and indeed the assistants were more numerous than the patients. The work is characterised by great thoroughness. The children are well provided with clothing and shoes, if necessary at the cost of the Government, and at the schools there is an interruption of the work for two hours in the middle of the day for food and rest and recreation, a condition which apparently does not obtain in America. In the working-man's museum great attention is paid to the illustration of methods which relate to the preservation of health and to the rearing of healthy children. Again, in the direction of disease prevention the streets are kept scrupulously clean, and the malignant fly is thus deprived of his opportunity. One of the results is seen in the absence of typhoid fever, of which, it is said, only a single case has been known in Munich during several years. This occurred in the house of a dairyman, who as a reward was sent to prison for nine years.

Professor Pirquet's Kinderklinik at Vienna is pronounced to be one of the best equipped and best organised children's hospitals in Europe. Clinical assistants must understand German and must undertake to stay for at least six months. Infectious diseases, such as diphtheria, measles, scarlet fever, and gonorrhœa, are treated in beds separated by glass partitions. Special attention is paid to tuberculosis, and both in winter and summer children are kept on the roof of the hospital, and in the warm weather the only clothing permitted to the child is a hat! The Pirquet test is used as a routine measure, as is also examination by the *x*-rays. Tuberculin treatment is given twice a week, and impressive results are seen in tuberculous peritonitis and adenitis, as well as in phthisis pulmonalis. Again, illustrations of hereditary syphilis are seen in great numbers. Good results are recorded of the effects of splenectomy in icterus hæmorrhagica, and in one case the red blood cells increased within three weeks after operation from 800,000 to over 4,000,000. One of the physicians treats pulmonary tuberculosis by suggestion and claims excellent results.

### Hindrances of the War.

At Berlin (visited two months after the commencement of the war) the most impressive thing is pronounced to be the infant feeding of Finkelstein and Meyer. Here and in the other clinics research and teaching work were in abeyance, as the assistants were engaged in work for the army. The hospitals are to a large extent given over to wounded soldiers, and only the most urgent cases of disease in children receive attention. Work has been in progress in such directions as the immunity of the newly born, the nature of diphtheria immunity, tuberculous disease of the intestine, the treatment of tuberculous meningitis, the cutaneous reaction of diphtheria, and research work with the electrocardiograph, but all these and others are in the meantime suspended. Professor Pirquet, too, has had to abandon much of his special work and is devoting himself to the wounded soldiers who are received into his hospital. Dr. Colliver expresses the opinion that even if the war stopped to-day it would take Germany and Austria more than a generation to regain the position they have so long held in the scientific world. He believes, also, that one of the effects of the war will be a tendency to shift this scientific and research spirit to America. Certainly his record of the confusions and hindrances which the war has created indicates how far-reaching and disastrous will be the effects both on scientific progress and on the practical methods by means of which the health of the rising generation are to be promoted. Science, it is said, knows no frontiers, but the international bitterness which will long survive the cessation of hostilities will seriously interfere with the freedom of communication upon which the diffusion of knowledge is largely dependent.

In a summary of his observations Dr. Colliver concludes as follows: Liverpool for orthopædics;

London for malnutrition, rheumatism, chorea, endocarditis, and rheumatic nodules; Munich for hygiene, prophylaxis, health regulations and food stations, and preservation of the normal; Vienna for tuberculosis, syphilis, $x$-rays, pathology, and research work; Berlin for infant feeding; and America for practicability and some of the best of all. It is sometimes said that travel in foreign countries has the advantage of making one more content with the state of affairs at home. The fine feathers of far-away birds are not always so enchanting when seen close at hand, and no one will grudge Dr. Colliver his conclusion that there are abundant good things in his own country. No doubt he will wish to make them still better, and in this ambition we cordially wish him every success.

